# Supplementary material for: Dissociable contributions of cortical thickness and surface area to cognitive ageing: evidence from multiple longitudinal cohorts
Source: bioRxiv. 2026 Mar 21:2026.03.20.713139. Preprint. [Version 1] doi: 10.64898/2026.03.20.713139 (PMC13015390; doi:10.64898/2026.03.20.713139)
Supplement: Supplement 1 [file media-1.pdf]

Supplementary Materials

Supplementary Table 1. Indirect effects of age and polygenic score on cognition through cortical thickness and surface area across cohorts with YoE.

Cohort	N	Path	Mediator	β	p	Effect %	Model comparison
**CamCAN**	621	Age → *g*	Thickness	0.07	.003	12%	$\Delta\chi^2$=44.00, p<.001
			Area	0.04	.002	7%	
		PGS → *g*	Thickness	0.01	.126	4%	
			Area	0.01	.011	11%	
		YoE → *g*	NA	0.28	<.001	NA	
**OASIS-3**	981	Age → *g*	Thickness	0.14	<.001	29%	$\Delta\chi^2$=32.97, p<.001
			Area	0.06	<.001	12%	
		YoE → *g*	NA	0.24	<.001	NA	
**HABS-HD**	2750	Age → *g*	Thickness	0.02	<.001	8%	$\Delta\chi^2$=98.66, p<.001
			Area	0.02	<.001	7%	
		YoE → *g*	NA	0.62	<.001	NA	

Supplementary Table 2. Correlated change results from simple models, with ('Preserved') and without ('Adjusted (gamm)') adjustments for non-linear age effects using a GAMM.

Cohort	N	Non-linear age effects	Thickness change	Area change	Steiger's test
**CamCAN**	131	Preserved	**0.17 (3.1%), p=.046**	0.01 (<1%), p=.936	z=1.34, p=.181
		Adjusted (GAMM)	0.11 (1.3%), p=.195	-0.05 (<1%), p=.582	z=1.24, p=.213
**OASIS-3**	511	Preserved	**0.18 (3.1%), p<.001**	0.04 (<1%), p=.383	**z=2.51, p=.012**
		Adjusted (GAMM)	**0.14 (1.9%), p=.002**	-0.01 (<1%), p=.742	**z=2.70, p=.007**
**HABS-HD**	1257	Preserved	**0.10 (1.1%), p<.001**	**0.06 (<1%), p=.034**	z=1.22, p=.224
		Adjusted (GAMM)	**0.11 (1.1%), p<.001**	0.04 (<1%), p = .125	z=1.70, p=.088

Supplementary Table 3. Correlated change and baseline Cortex – cognition change associations from extended longitudinal models, estimated with and without adjustment for non-linear age effects. Linear Hypothesis Test (LHT) results indicate whether change–change associations differ significantly between cortical thickness and surface area.

	N	Non-linear age effects	Thickness change	Thickness baseline	Area change	Area baseline	LHT
**CamCAN**	131	Preserved	**0.18 (3.4%), p=.037**	**0.24 (6%), p=.005**	0.004 (<1%), p=.964	0.08 (<1%) p=.335	F=2.09, p=.150
		Adjusted (gamm)	0.12 (1.5%), p=.167	0.10 (<1%), p=.282	-0.03 (<1%), p=.701	0.03 (<1%), p=.682	F=1.70, p=.195
**OASIS-3**	511	Preserved	**0.18 (3.4%), p<.001**	**0.23 (5.5%), p<.001**	-0.01 (<1%), p=.766	-0.02 (<1%), p=.576	**F=8.33, p=.004**
		Adjusted (gamm)	**0.16 (2.5%), p<.001**	**0.17 (2.9%), p<.001**	-0.05 (<1%), p=.260	-0.05 (<1%), p=.268	**F=9.52, p=.002**
**HABS-HD**	1257	Preserved	**0.13 (1.6%), p <.001**	**0.14 (1.9%), p<.001**	0.04 (<1%), p=.214	-0.01 (<1%), p=.604	**F=4.89, p=.027**
		Adjusted (gamm)	**0.13 (1.6%), p<.001**	**0.13 (1.5%), p<.001**	0.02 (<1%), p=.421	-0.01 (<1%), p=.676	**F = 6.42, p = .011**

Supplementary Table 4. Bivariate latent change score model results for latent change covariance and cross-lagged relationships between baseline and change measure without (top) and with (bottom) adjustment for baseline age (dg ~ A0 & dThK or dArea ~ A0).

Cohort	N	Measure	dCortex–d*g*	Cortex–d*g*	g–dCortex	dCortex–A0	d*g*–A0
**CamCAN**	131	Thickness	0.13, p=.149	0.04, p=.662	-0.02, p.806	**-0.31, p=.003**	**-0.45, p<.001**
		Area	-0.04, p=.720	0.09, p=.131	0.07, p=.447	-0.05, p =.612	**-0.46, p<.001**
**OASIS-3**	137	Thickness	**0.25, p=.003**	**0.19, p=.020**	0.152, p=.053	-0.21, p=.018	0.06, p=.474
		Area	-0.009, p=.909	0.01, p=.889	0.11, p=.223	**-0.29, p<.001**	-0.04, p=.624
**HABS-HD**	533	Thickness	**0.16, p<.001**	0.03, p=.479	**0.10, p=.013**	**-0.13, p<.001**	**-0.21, p<.001**
		Area	**0.11, p=.010**	-0.01, p=.807	**0.13, p=.003**	**-0.02, p=.003**	**-0.22, p<.001**

Supplementary Table 5. Latent growth model results for latent change covariance and cross-lagged relationships between baseline and change. sCortex – latent slope for morphometric feature, iCortex – intercept (baseline) for morphometric feature; sg – latent slope for cognition, ig – intercept for cognition.*

**Cohort**	**N**	**Measure**	**sCortex–s*g***	**iCortex–s*g***	**i*g*–sCor**
**OASIS-3**	137	Thickness	**0.25, p=.013**	**0.21, p=.012**	**0.28, p<.001**
		Area	-0.15, p=.944	0.04, p=.421	**0.39, p=.038**
**HABS-HD**	533	Thickness	**0.20, p<.001**	**0.12, p=.007**	**0.10, p=.026**
		Area	**0.22, p=.002**	-0.04, p=.329	**0.20, p=.002**

** The variances of observed measures were constrained to a small value (0.01) to correct for occasional negative variances (so-called "Heywood cases", a problem and solution both common in latent growth modelling of behavioural constructs).*

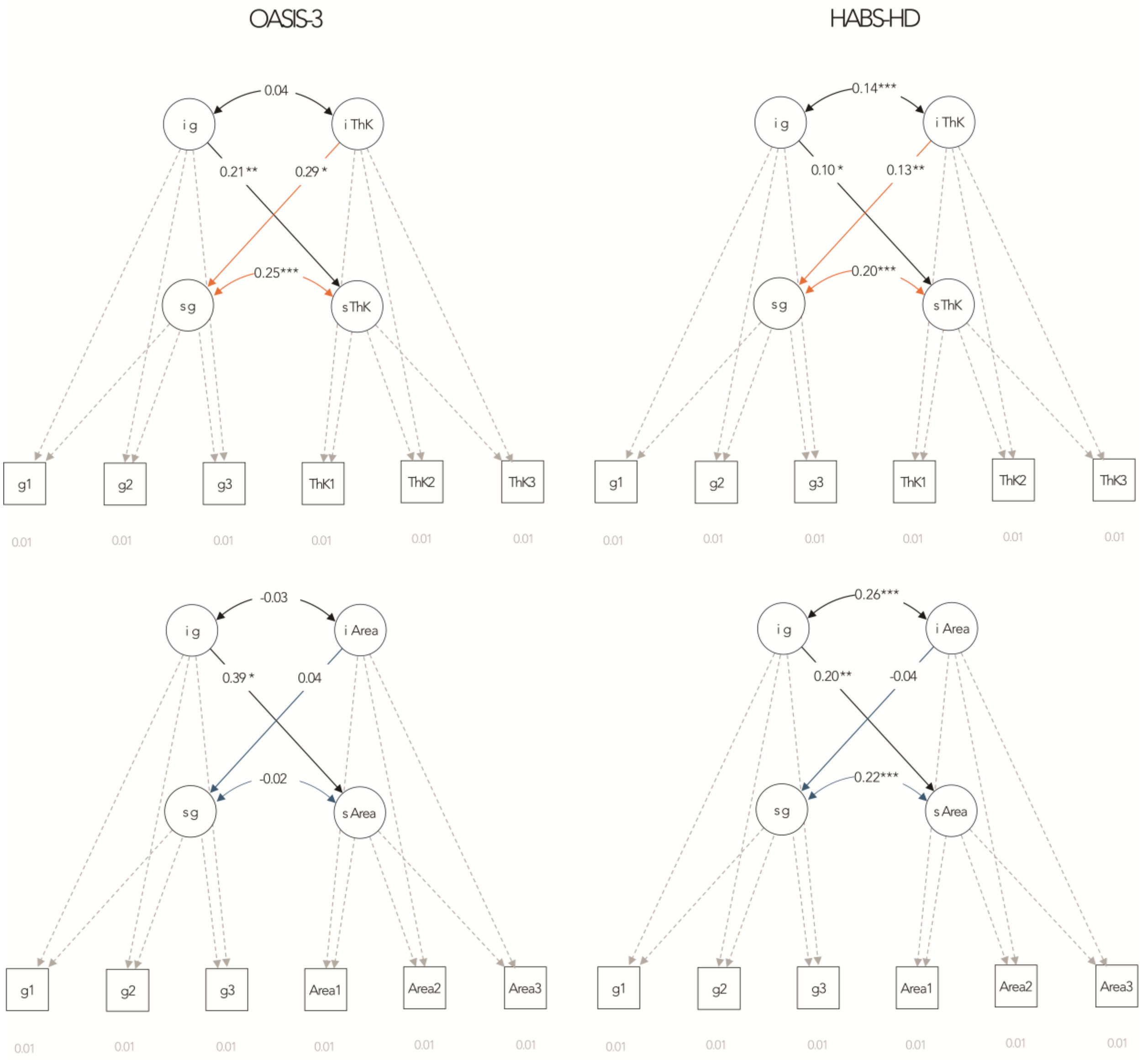

Supplementary Figure 1. Visual representation of latent growth models in the OASIS-3 and HABS-HD cohorts. Circles denote latent variables: baseline (intercept) levels (i g; i ThK (thickness) / i Area) and latent change factors, i.e. 'slopes' (s g; s ThK / s Area). Rectangles represent observed indicators of g at selected three time points (g1, g2, g3) and cortical metrics (Thk1-Thk3; Area1-Area3). Straight single-headed arrows indicate regression paths; curved double-headed arrows indicate covariances. Dashed paths represent parameters fixed for model identification. Coloured paths show the cross-domain coupling effects of primary interest (orange: thickness models; turquoise: area models). Values are standardised parameter estimates. Statistical significance is indicated as: $p < .05$ (), $p < .01$ (), $p < .001$ ().

Supplementary Table 6. Region of Interest (ROI) associations between cortical thickness or surface area change and cognitive change across 34 Desikan–Killiany cortical regions (collapsed across hemisphere).ROIs that survived FDR correction are in bold. ROIs highlighted in grey (first three rows) were those tested by Nyberg et al. (2023). Note that regional area measures were not available in the OASIS-3 cohort.

Cohort	CamCAN (n=131)				OASIS-3 (n=511)			HABS-HD (n= 1257)			
Measure	Thickness		Area		Thickness		Area	Thickness		Area	
ROI	β	p	β	p	β	p	β, p	β	p	β	p
inferiorparietal	0.11	.203	0.08	.782	0.09	.036	NA	**0.07**	**.012**	0.02	.412
middletemporal	0.07	.399	0.15	.493	**0.17**	**<.001**	NA	**0.09**	**.002**	0.06	.029
rostralmiddlefrontal	0.04	.620	-0.05	.823	0.06	.195	NA	0.04	.171	0.06	.031
bankssts	0.03	.693	0.00	.973	0.07	.099	NA	0.04	.125	0.03	.220
caudalanteriorcingulate	-0.03	.717	0.10	.606	0.02	.610	NA	0.04	.169	0.02	.412
caudalmiddlefrontal	0.07	.445	-0.10	.579	0.00	.997	NA	**0.07**	**.009**	0.05	.064
cuneus	0.17	.052	0.05	.823	0.09	.046	NA	0.05	.068	0.02	.529
entorhinal	0.21	.014	-0.16	.493	**0.18**	**<.001**	NA	**0.08**	**.003**	0.01	.726
frontalpole	-0.01	.911	0.11	.579	0.06	.182	NA	0.02	.472	0.03	.299
fusiform	0.14	.102	0.08	.735	0.08	.076	NA	**0.12**	**<.001**	-0.03	.319
inferiortemporal	0.11	.229	0.05	.823	0.11	.012	NA	**0.10**	**<.001**	-0.01	.857
insula	0.06	.500	0.11	.579	0.09	.035	NA	**0.09**	**.001**	0.04	.203
isthmuscingulate	-0.03	.721	0.12	.579	**0.16**	**<.001**	NA	0.04	.151	0.04	.144
lateraloccipital	0.23	.009	0.01	.973	0.10	.032	NA	0.03	.264	0.03	.322
lateralorbitofrontal	0.05	.557	0.04	.855	0.05	.266	NA	0.03	.376	0.07	.019
lingual	0.22	.011	0.01	.973	0.07	.102	NA	0.01	.697	0.07	.011
medialorbitofrontal	0.04	.626	0.14	.551	0.05	.289	NA	0.02	.533	0.03	.274
paracentral	0.14	.102	-0.17	.493	0.00	.912	NA	**0.07**	**.013**	0.00	.957
parahippocampal	0.17	.055	0.11	.579	**0.19**	**<.001**	NA	**0.12**	**<.001**	0.05	.065
parsopercularis	0.02	.783	-0.03	.918	0.03	.499	NA	0.05	.061	0.05	.101
parsorbitalis	-0.11	.194	0.20	.493	0.00	.943	NA	0.05	.087	0.01	.612
parstriangularis	0.10	.279	0.05	.823	0.04	.349	NA	0.04	.137	0.05	.061
pericalcarine	0.21	.016	-0.01	.973	0.05	.304	NA	0.02	.541	0.03	.374
postcentral	0.20	.025	-0.18	.493	0.01	.752	NA	0.06	.024	-0.02	.516
posteriorcingulate	-0.07	.423	0.15	.493	0.11	.013	NA	0.06	.033	0.04	.115
precentral	0.14	.112	-0.07	.795	0.04	.368	NA	**0.10**	**.001**	0.01	.825
precuneus	0.19	.032	0.02	.973	0.08	.084	NA	**0.07**	**.012**	0.06	.032
rostralanteriorcingulate	-0.06	.530	0.04	.855	0.09	.037	NA	0.06	.033	0.03	.249
superiorfrontal	0.06	.525	-0.13	.578	0.05	.290	NA	**0.09**	**.001**	0.05	.089
superiorparietal	0.19	.034	0.00	.973	0.08	.079	NA	0.06	.039	0.02	.516
superiortemporal	0.17	.049	-0.01	.973	**0.14**	**.002**	NA	**0.11**	**<.001**	0.01	.631
supramarginal	0.15	.093	-0.07	.795	0.08	.085	NA	0.06	.035	0.04	.147
temporalpole	0.17	.054	0.11	.579	0.11	.015	NA	**0.11**	**<.001**	0.05	.095
transversetemporal	0.19	.031	0.06	.823	0.07	.113	NA	0.03	.321	0.00	.890

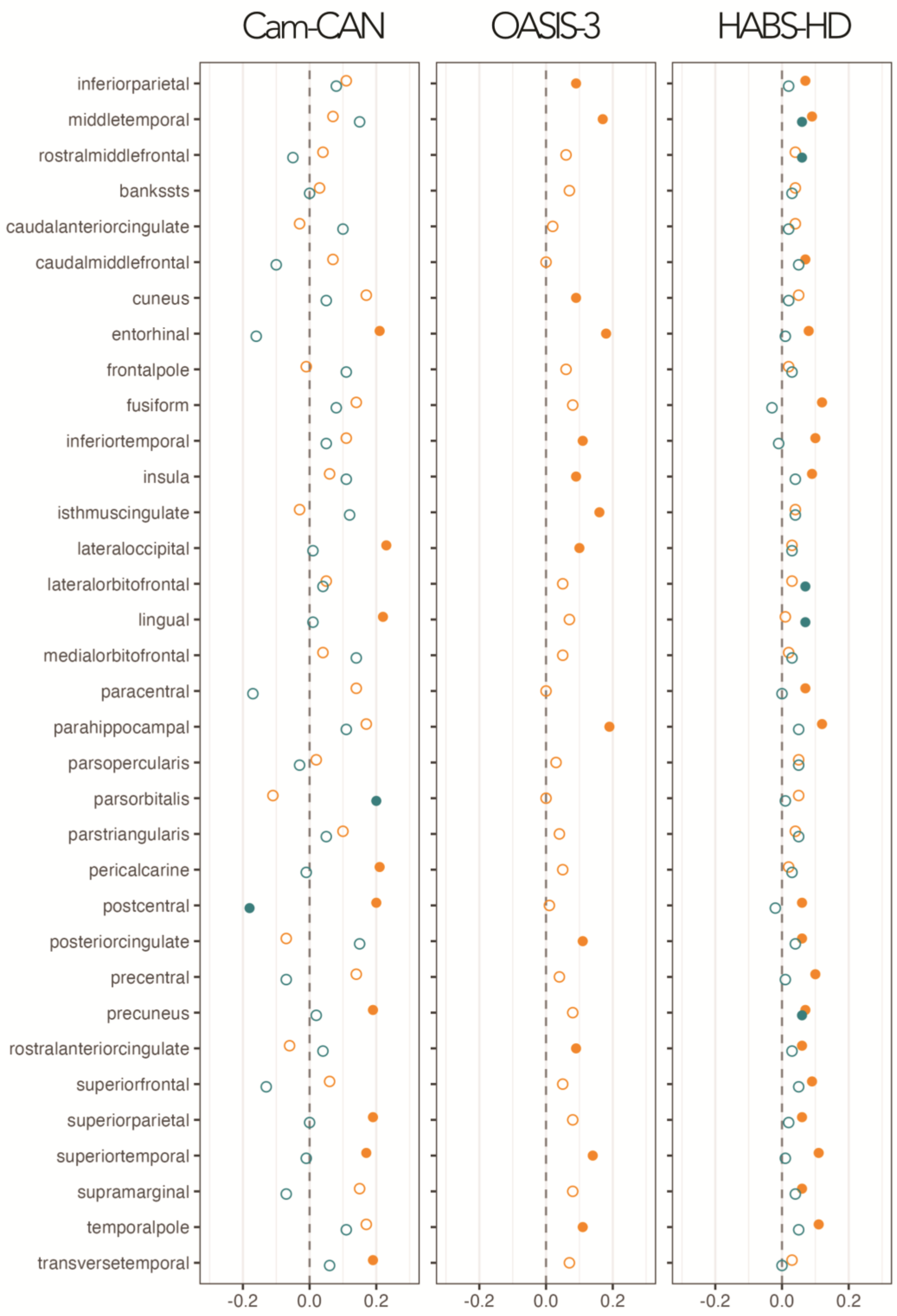

Supplementary Figure 2. Forest plots showing ROI-level thickness-g (orange) and area-g (turquoise) longitudinal coupling across Desikan–Killiany regions.
